# Supplementary material for: Quantification of H3.1-nucleosomes using a chemiluminescent immunoassay: A reliable method for neutrophil extracellular trap detection
Source: PLoS One. 2025 Aug 6;20(8):e0329352. doi: 10.1371/journal.pone.0329352 (PMC12327617; doi:10.1371/journal.pone.0329352)
Supplement: S1 Fig — Neutrophil-like DMSO-differentiated HL-60 cells were treated during 5 hours with the NETosis-inducer PMA (PMA, lower panel), or not treated (DMSO, upper panel). DNA and membranes were stained with DAPI (Blue) and Cell Mask Orange (Orange-Red), respectively. Scale bars 50 µm. (PDF) [file pone.0329352.s001.pdf]

S1 Figure: PMA treatment induces NETs formation in DMSO-differentiated HL-60 cells

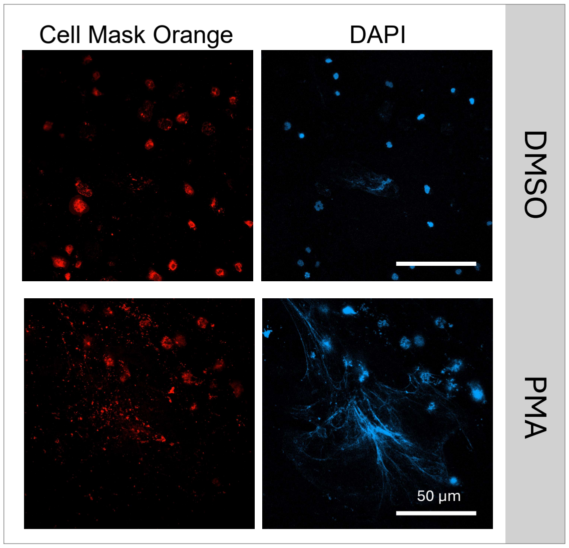

Supplementary Figure 1 : Neutrophil-like DMSO-differentiated HL-60 cells were treated during 5 hours with the NETosis-inducer PMA (PMA, lower panel), or not treated (DMSO, upper panel). Membrane and DNA were stained with Cell Mask Orange (Orange-Red) and DAPI (Blue), respectively. Scale bars 50 µm.
