# Supplementary material for: Quantification of H3.1-nucleosomes using a chemiluminescent immunoassay: A reliable method for neutrophil extracellular trap detection
Source: PLoS One. 2025 Aug 6;20(8):e0329352. doi: 10.1371/journal.pone.0329352 (PMC12327617; doi:10.1371/journal.pone.0329352)
Supplement: S2 Fig — Neutrophil-like DMSO-differentiated HL-60 cells (A-B) or primary neutrophils isolated from human whole blood (C-D) treated with Calcium Ionophore A23187 were stained with anti-MPO (A and B, purple; C, yellow), anti-histone H3.1 (A, C and D, green), anti-nucleosome (B, green) or anti-H3Cit (D, yellow) antibodies. DNA and membranes were stained with DAPI (blue) and Cell MaskTM Orange (orange-red), respectively. Scale bars, 20µm.DMSO, dimethyl sulfoxide; CI, calcium ionophore A23187; MPO, myeloperoxidase; NETs, neutrophil extracellular traps. (PDF) [file pone.0329352.s002.pdf]

S2 Figure. Immunostaining of NETs derived from HL-60 cells or primary neutrophils treated with calcium ionophore A23187 demonstrates the presence of H3.1-nucleosomes

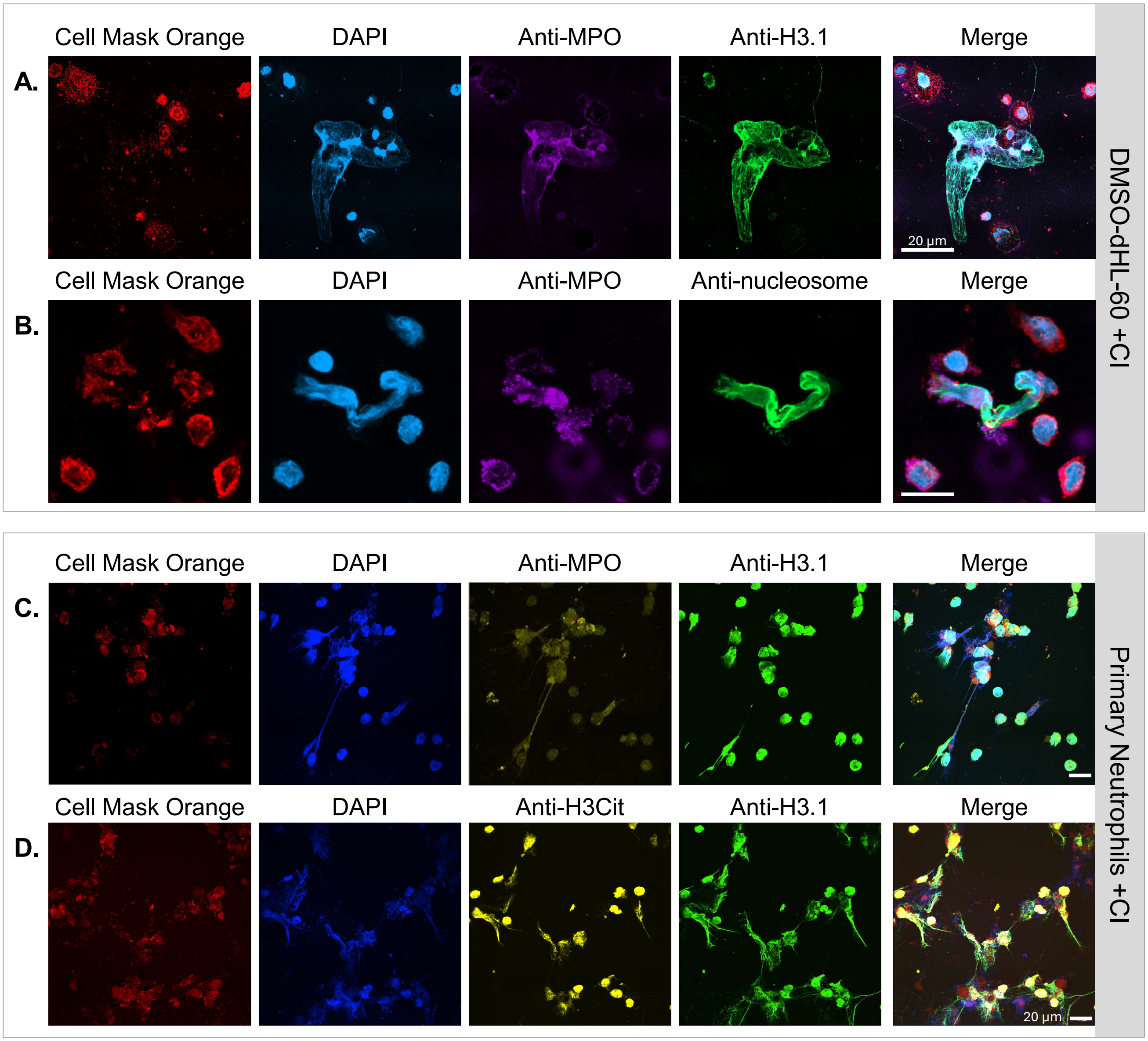

Supplementary Figure 2 : Neutrophil-like DMSO-differentiated HL-60 cells (A-B) or primary neutrophils isolated from human whole blood (C-D) treated with Calcium Ionophore A23187 were stained with anti-MPO (A and B, purple; C, yellow), anti-histone H3.1 (A, C and D, green), anti-nucleosome (B, green) or anti-H3Cit (D, yellow) antibodies. DNA and membranes were stained with DAPI (blue) and Cell Mask^{TM} Orange (orange-red), respectively. Scale bars, 20µm.

DMSO, dimethyl sulfoxide; CI, calcium ionophore A23187; MPO, myeloperoxidase; NETs, neutrophil extracellular traps.
