# Supplementary material for: Quantification of H3.1-nucleosomes using a chemiluminescent immunoassay: A reliable method for neutrophil extracellular trap detection
Source: PLoS One. 2025 Aug 6;20(8):e0329352. doi: 10.1371/journal.pone.0329352 (PMC12327617; doi:10.1371/journal.pone.0329352)
Supplement: S3 Fig — Quantification of H3.1-, H3R8Cit-, and H3R2,8,17Cit-recombinant nucleosomes using the H3.1-nucleosome immunoassay. Results are expressed as a percentage relative to the theoretical concentration of nucleosomes loaded in the samples (% recovery). (PDF) [file pone.0329352.s003.pdf]

S3 Figure: Detection of citrullinated H3.1-nucleosomes using the H3.1-nucleosome immunoassay

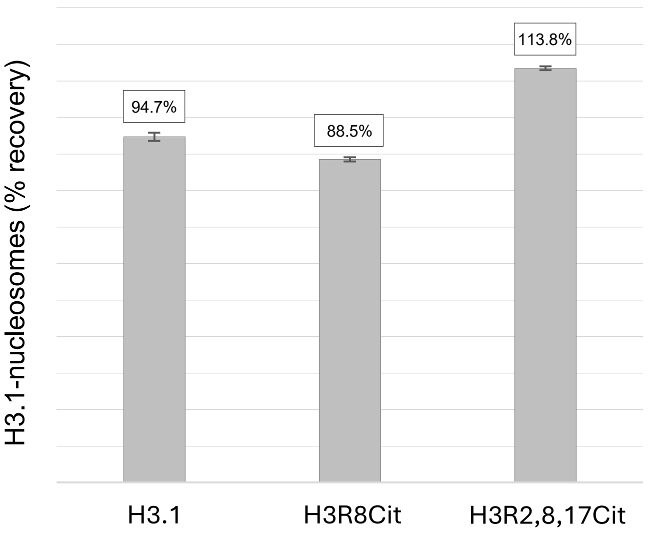

Supplementary Figure 3: Quantification of H3.1-, H3R8Cit-, and H3R2,8,17Cit-recombinant nucleosomes using the H3.1-nucleosome immunoassay. Results are expressed as a percentage relative to the theoretical concentration of nucleosomes loaded in the samples (% recovery).
