# Supplementary material for: Quantification of H3.1-nucleosomes using a chemiluminescent immunoassay: A reliable method for neutrophil extracellular trap detection
Source: PLoS One. 2025 Aug 6;20(8):e0329352. doi: 10.1371/journal.pone.0329352 (PMC12327617; doi:10.1371/journal.pone.0329352)
Supplement: S4 Fig — Panels A to D present box plot analysis of H3.1-nucleosome levels (A; ng/mL), H3Cit-nucleosome levels (B; ng/mL), MPO-DNA complex levels (C; ODs), H3Cit-DNA complex levels (D, ng/mL) in control patients and in patients suffering of NETs-related diseases (Sepsis and COVID-19). The whiskers represent the 25th–75th percentile with median. **** represent p-value <0.0001, calculated using the Mann–Whitney U test. Panels E to H present ROC curve analysis of circulating H3.1-nucleosomes (E), H3Cit-nucleosomes (F), MPO-DNA complexes (G) and H3Cit-DNA complexes (H) for discrimination of control versus NETs samples. AUC, area under the curve. (PDF) [file pone.0329352.s004.pdf]

S4 Figure. Comparative measures of H3.1-nucleosomes, H3Cit-nucleosomes, MPO-DNA, and H3Cit-DNA complexes in plasma from control individuals and patients with NETs-related pathologies

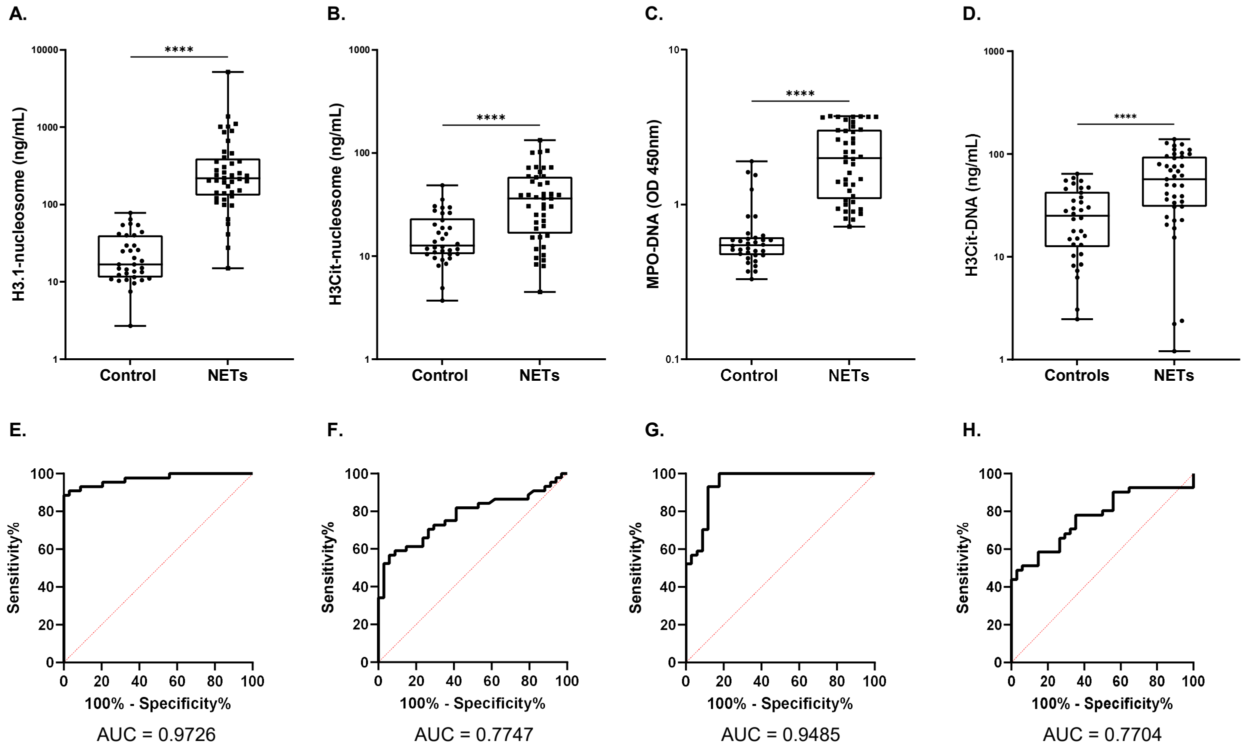

Supplementary Figure 4 : Panels A to D present box plot analysis of H3.1-nucleosome levels (A; ng/mL), H3Cit-nucleosome levels (B; ng/mL), MPO-DNA complex levels (C; ODs), H3Cit-DNA complex levels (D, ng/mL) in control patients and in patients suffering of NETs-related diseases (Sepsis and COVID-19). The whiskers represent the 25th–75th percentile with median. **** represent p-value <0.0001, calculated using the Mann–Whitney U test. **Panels E to H present** ROC curve analysis of circulating H3.1-nucleosomes (E), H3Cit-nucleosomes (F), MPO-DNA complexes (G) and H3Cit-DNA complexes (H) for discrimination of control versus NETs samples.

AUC, area under the curve.
