# Supplementary material for: Quantification of H3.1-nucleosomes using a chemiluminescent immunoassay: A reliable method for neutrophil extracellular trap detection
Source: PLoS One. 2025 Aug 6;20(8):e0329352. doi: 10.1371/journal.pone.0329352 (PMC12327617; doi:10.1371/journal.pone.0329352)
Supplement: S1 Table — H3.1-nucleosomes levels, expressed in ng/mL, indicate the depletion of nucleosomes after immunoprecipitation (IP) in comparison to the level present in the initial samples (HL-60 NETs unprocessed). Depletion results are expressed in %.NETs, neutrophil extracellular traps. (PDF) [file pone.0329352.s005.pdf]

S1 Table: H3.1-nucleosomes depletion after immunoprecipitation

Samples	H3.1-nucleosome concentration post IP (ng/mL)	% depletion
HL-60 NETs unprocessed	468.4	-
IP anti-nucleosome	12.5	97%
IP anti-H3.1	5.7	99%
IP anti-MPO	328.0	30%
IP IgG2a isotype	458.1	2%
IP rabbit IgG isotype	490.7	-5%

Supplementary Table 1: H3.1-nucleosome levels, expressed in ng/mL, showing the depletion of H3.1-nucleosomes after immunoprecipitation in comparison to the level present in the initial samples (dHL-60 NETs unprocessed). Depletion results are expressed in %.
