# Supplementary material for: Quantification of H3.1-nucleosomes using a chemiluminescent immunoassay: A reliable method for neutrophil extracellular trap detection
Source: PLoS One. 2025 Aug 6;20(8):e0329352. doi: 10.1371/journal.pone.0329352 (PMC12327617; doi:10.1371/journal.pone.0329352)
Supplement: S2 Table — NETs, neutrophil extracellular traps; Mean cc, mean of H3.1-nucleosomes assay concentration expressed in ng/mL. (PDF) [file pone.0329352.s006.pdf]

S2 Table: H3.1-nucleosome immunoassay linearity

	Mix 1				Mix 2				Mix 3			
	Expected value	Mean cc	Linear fit	Non linearity	Expected value	Mean cc	Linear fit	Non linearity	Expected value	Mean cc	Linear fit	Non linearity
**Sample 1 (S1)**	**5.2**	5.2	4.6	**12.9%**	**115.8**	115.8	94.4	**22.7%**	**389.8**	389.8	376.7	**3.5%**
**90% S1 - 10% S2**	26.3	25.4	25.8	**-1.8%**	201.4	180.2	180.7	**-0.3%**	472.2	464.5	460.5	**0.9%**
**80% S1 - 20% S2**	47.3	43.3	47.0	**-7.9%**	287.0	262.6	266.9	**-1.6%**	554.7	543.0	544.3	**-0.2%**
**70% S1 - 30% S2**	68.4	66.2	68.3	**-3.1%**	372.6	338.5	353.2	**-4.2%**	637.2	626.7	628.1	**-0.2%**
**60% S1 - 40% S2**	89.5	90.0	89.5	**0.6%**	458.2	421.8	439.5	**-4.0%**	719.6	702.3	711.9	**-1.3%**
**50% S1 - 50% S2**	110.5	107.0	110.7	**-3.4%**	543.8	519.6	525.8	**-1.2%**	802.1	770.0	795.7	**-3.2%**
**40% S1 - 60% S2**	131.6	132.5	131.9	**0.4%**	629.4	617.8	612.1	**0.9%**	884.5	880.0	879.5	**0.1%**
**30% S1 - 70% S2**	152.7	149.5	153.2	**-2.4%**	715.0	700.1	698.4	**0.2%**	967.0	972.5	963.3	**1.0%**
**20% S1 - 80% S2**	173.7	177.2	174.4	**1.6%**	800.6	800.3	784.7	**2.0%**	1049.4	1036.0	1047.1	**-1.1%**
**10% S1 - 90% S2**	194.8	199.7	195.6	**2.1%**	886.2	881.5	871.0	**1.2%**	1131.9	1139.3	1130.8	**0.7%**
**Sample 2 (S2)**	**215.9**	215.9	216.8	**-0.5%**	**971.8**	971.8	957.3	**1.5%**	**1214.4**	1214.4	1214.6	**0.0%**
