# Supplementary material for: Quantification of H3.1-nucleosomes using a chemiluminescent immunoassay: A reliable method for neutrophil extracellular trap detection
Source: PLoS One. 2025 Aug 6;20(8):e0329352. doi: 10.1371/journal.pone.0329352 (PMC12327617; doi:10.1371/journal.pone.0329352)
Supplement: S3 Table — NETs, neutrophil extracellular traps. (PDF) [file pone.0329352.s007.pdf]

S3 Table: H3.1-nucleosome immunoassay interferences and cross-reactivity

Potentially interfering agent	Threshold concentration
Haemoglobin	50 mg/dL
Bilirubin conjugate	20 mg/dL
Bilirubin non conjugated	20 mg/dL
Protein	9 g/dL
Triglyceride (intralipid)	3000 mg/dL
Cholesterol	300 mg/dL
HAMA (Human anti-mouse antibodies)	600 ng/mL
Rheumatoid Factor	2940 IU/mL
