# Supplementary material for: Quantification of H3.1-nucleosomes using a chemiluminescent immunoassay: A reliable method for neutrophil extracellular trap detection
Source: PLoS One. 2025 Aug 6;20(8):e0329352. doi: 10.1371/journal.pone.0329352 (PMC12327617; doi:10.1371/journal.pone.0329352)
Supplement: S4 Table — NETs, neutrophil extracellular traps; ROC, receiver operating characteristic. (PDF) [file pone.0329352.s008.pdf]

S4 Table: Descriptive statistics

	Control donors	« NETs » patients
**Number of samples**	236	302
**Descriptive statistics (Nu.Q® H3.1 – ng/mL)**		
Minimum	3.26	5.89
25% Percentile	12.02	78.07
Median	22.81	242.1
Mean	30.66	721
75% Percentile	35	751.3
Maximum	175.9	6000
Range	172.7	5994
**95% Confidence interval of median**		
Actual confidence level	95.66%	95.62%
Lower confidence limit	19.57	173.6
Upper confidence limit	24.45	308.8
**Area under the ROC curve**	0.9193	
Std. Error	0.01174	
95% confidence interval	0.8963 to 0.9423	
P-value	<0.0001	
**Threshold at 65 ng/mL**		
Sensitivity %	79.14%	
Specificity %	91.53%	
