# Supplementary material for: Quantification of H3.1-nucleosomes using a chemiluminescent immunoassay: A reliable method for neutrophil extracellular trap detection
Source: PLoS One. 2025 Aug 6;20(8):e0329352. doi: 10.1371/journal.pone.0329352 (PMC12327617; doi:10.1371/journal.pone.0329352)
Supplement: S1 Raw Images — (PDF) [file pone.0329352.s009.pdf]

Western blot anti-MPO (Figure 2A)

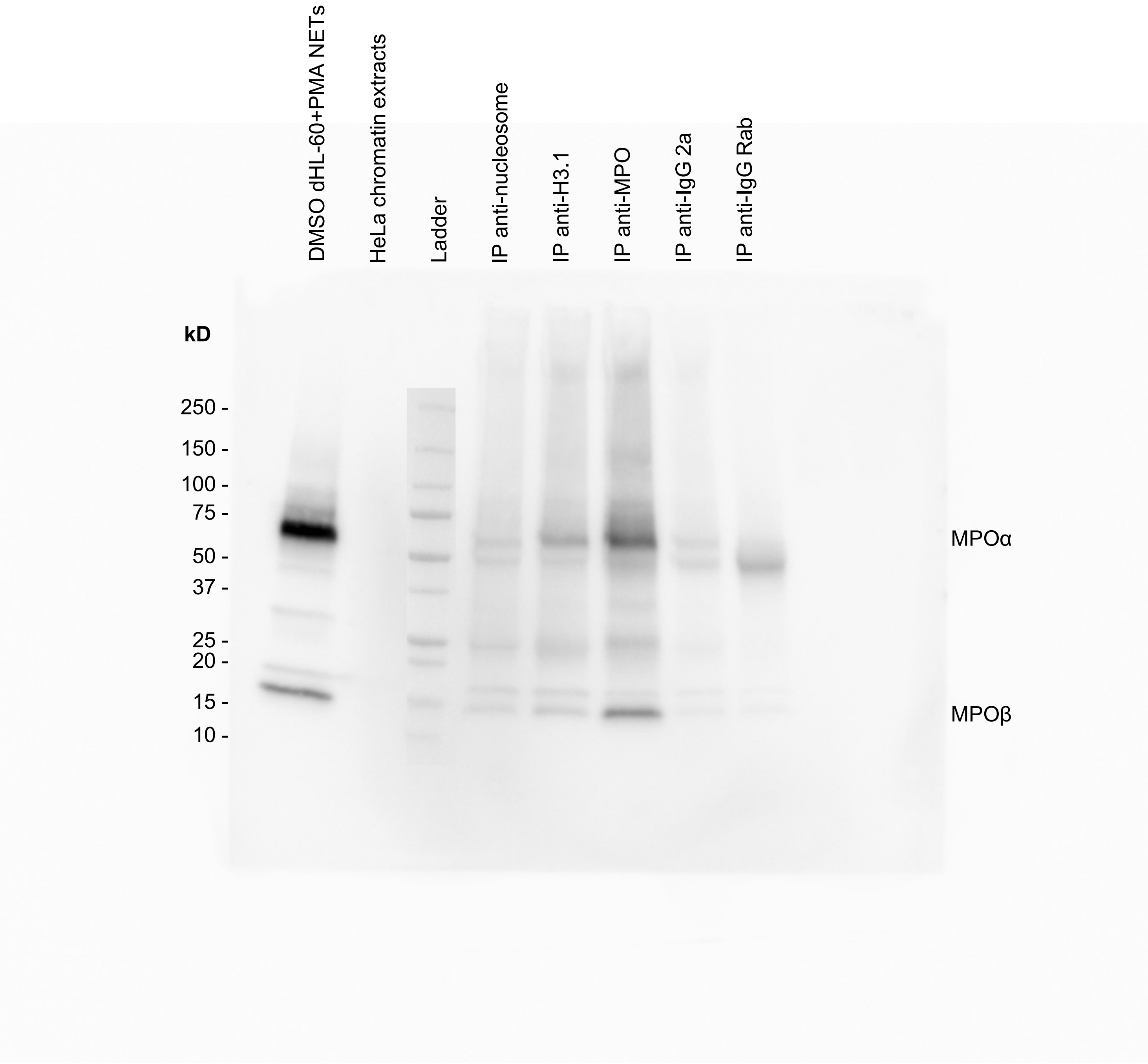

Primary Ab: MPO Merck #475915
Secondary Ab: VeriBlot Reagent (HRP) Abcam #ab131366
Exposure: Vilber Fusion-FX6 Instrument, chemiluminsecence, 1 sec.

Western blot anti-H4 (Figure 2A)

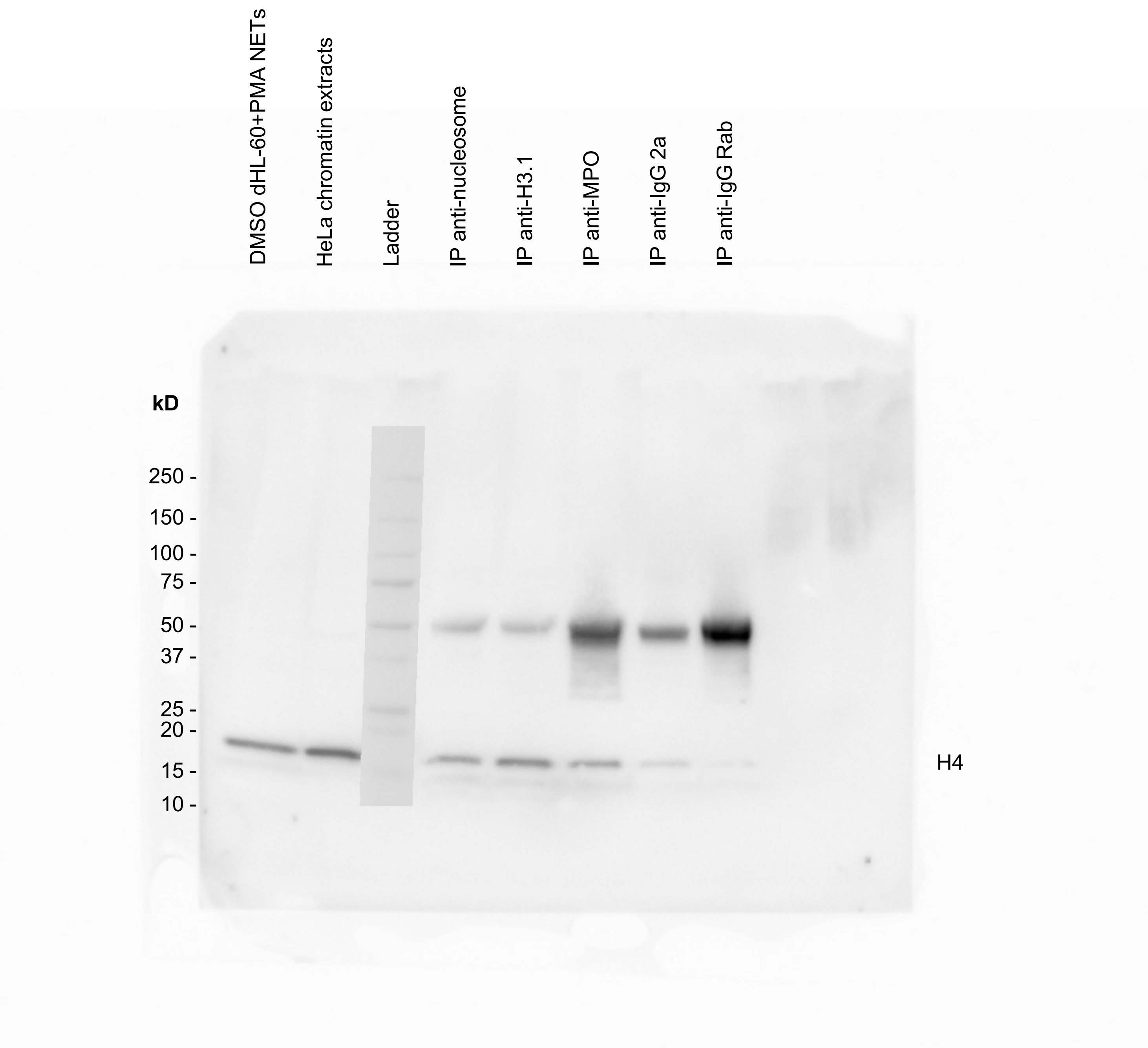

Primary Ab: histone H4, Abcam #ab177840
Secondary Ab: VeriBlot Reagent (HRP) Abcam #ab131366
Exposure: Vilber Fusion-FX6 Instrument, chemiluminsecence, 4.7 sec.

Western blot anti-H3 (Figure 2A)

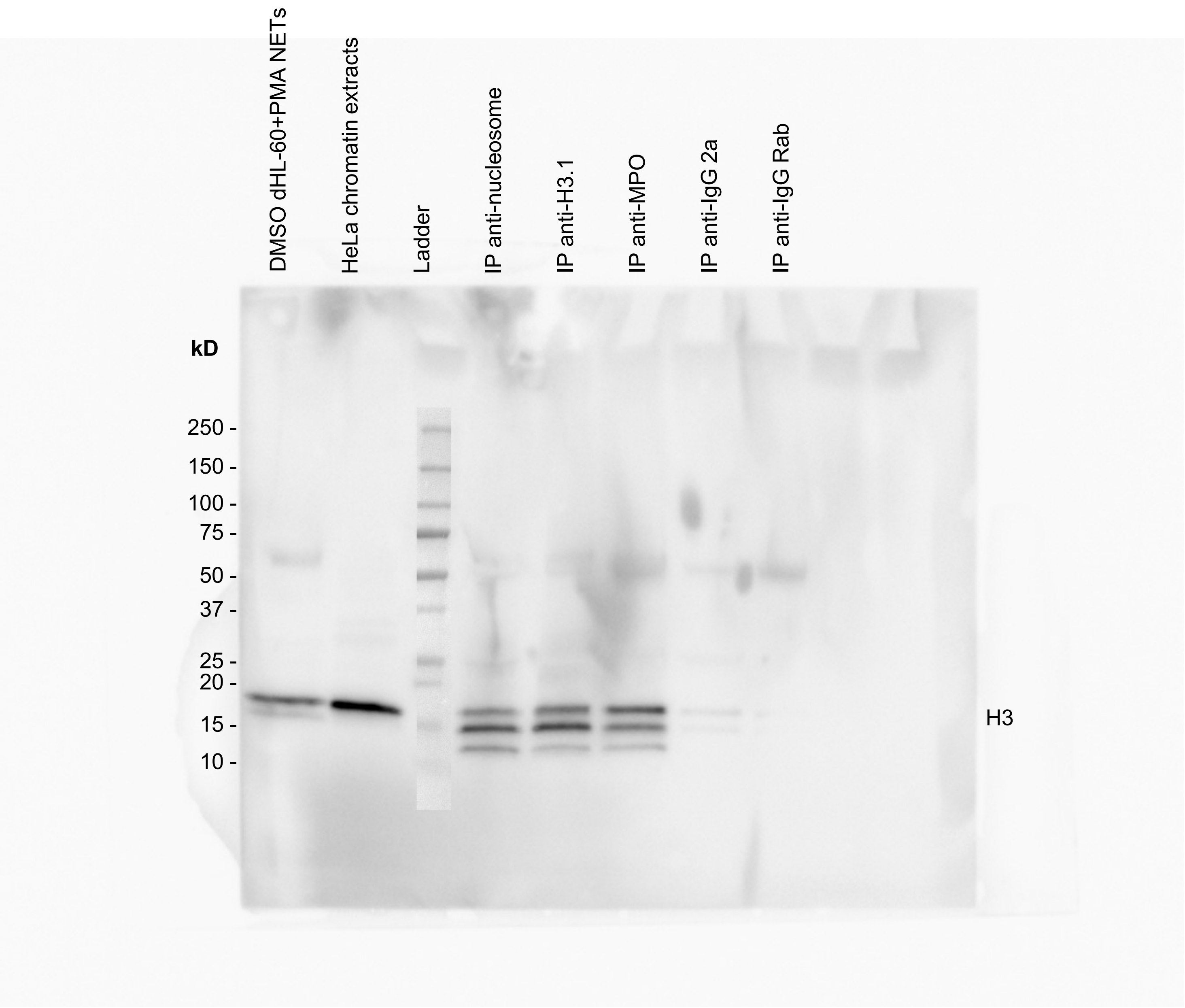

Primary Ab: histone H3 C-terminal, Active Motif #91297
Secondary Ab: VeriBlot Reagent (HRP) Abcam #ab131366
Exposure: Vilber Fusion-FX6 Instrument, chemiluminsecence, 1 sec.
